# GTP-binding protein Di-RAS3 diminishes the migration and invasion of non-small cell lung cancer by inhibiting the RAS/extracellular-regulated kinase pathway

**DOI:** 10.1080/21655979.2022.2031671

**Published:** 2022-02-16

**Authors:** Peng Kuang, An Xie, Jianxiong Deng, Jiaming Tang, Peijun Wang, Feng Yu

**Affiliations:** a Department of Oncology, The First Affiliated Hospital of Nanchang University, Nanchang, China; b Jiangxi Institute of Urology, The First Affiliated Hospital of Nanchang University, China

**Keywords:** Non-small cell lung cancer, DIRAS3, RAS/ERK pathway, metastasis, migration, invasion

## Abstract

The GTP-binding protein Di-Ras3 (DIRAS3) has been established as a maternally imprinted tumor suppressor gene. Growing evidence has correlated the DIRAS3 gene with tumor progression, but its role in non-small cell lung cancer (NSCLC) is rarely reported. Accordingly, the current study sought to evaluate the role and mechanism of DIRAS3 in NSCLC cell progression. First, we uncovered that DIRAS3 was poorly expressed in NSCLC tissues and cells. Subsequently, we examined the effect of DIRAS3 over-expression or knockdown in different lung cancer cells on their malignant phenotypes, with the help of transwell cell migration and invasion assays, and Western blot analyses. It was found that the over-expression of DIRAS3 inhibited the migration and invasion of A549 cells or H520 cells, whereas knockdown of DIRAS3 led to opposing trends. In addition, over-expression of DIRAS3 attenuated the tumor growth and reduced the number of lung tumor nodules. Mechanistically, DIRAS3 may inhibit the migration and invasion of NSCLC cells by inhibiting the RAS/extracellular-regulated kinase (ERK) signaling pathway. Collectively, our findings indicate that DIRAS3 could serve as a potential therapeutic target biomarker for NSCLC.

## Graphical abstract

**Figure:**
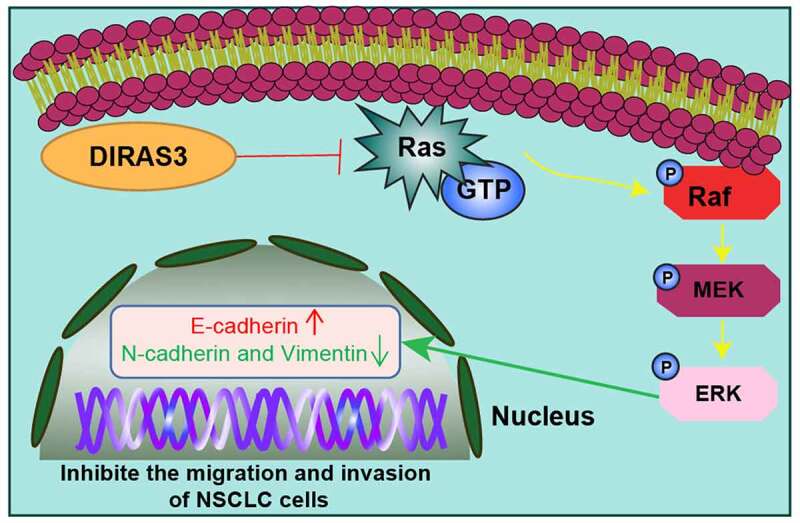

## Introduction

Lung cancer refers to tumors originating in the lung parenchyma or within bronchi, and further consists of two primary subtypes, namely small cell lung carcinoma and non-small cell lung cancer (NSCLC) [1]. Meanwhile, NSCLC represents the most common type of lung cancer, accounting for more than 80% of all lung cancer diagnoses [2]. The hard-done work of our peers has further shown that NSCLC is precipitated by chemical and physical mutagens-induced multistep carcinogenesis and can also develop due to a single-gene abnormality [3]. Moreover, numerous advances have been made in the field of NSCLC therapy owing to an elaborate understanding of its pathogenic genomic alterations; the application of new drugs and biomarkers could help to identify those patients who are more likely to react to immune checkpoint blockade therapy [4]. However, NSCLC patients still present with poor prognoses despite the tangible advances in early detection and development of novel chemotherapeutic regimens, such that the five-year survival rate is a measly 16% [5,6]. Consequently, it is of great interest to further advance the search for novel therapies against NSCLC.

The GTP-binding protein Di-RAS3 (DIRAS3), a member of the Ras superfamily, is known to be poorly expressed in a plethora of malignancies, including ovarian, breast, and hepatocellular carcinomas. What is more, up-regulation of DIRAS3 has been previously documented to restrict tumor growth, and further promote autophagy and tumor dormancy in the aforementioned cancers [7–9]. In addition, DIRAS3 is capable of suppressing cell migration and inducing autophagy in ovarian and breast cancers [10–12]. Further piquing our interest, a number of prior studies have suggested that DIRAS3 expression is negatively associated with cell survival of cancers, such that its expression could potentially restrict proliferation, foci formation, as well as invasiveness in culture [13,14]. Additionally, Field *et al*. previously reported that the tumor-suppressing gene (aplysia ras homolog I, ARHI, another name of DIRAS3) was highly methylated in the squamous cell lung carcinoma tissues [15]. Interestingly, the over-expression of the ARHI gene has been documented to be capable of inhibiting lung cancer cell growth and inducing apoptosis [16]. Nevertheless, the underlying mechanism of action of DIRAS3 in NSCLC cell migration and invasion is poorly defined. Furthermore, DIRAS3 (ARHI) encodes a 26 kDa GTPase with 50–60% homology to Ras and Rap, with the main difference being the increased 34 amino acid N-terminal extension required for majority of DIRAS3 functioning [17]. Meanwhile, the aforementioned RAS-RAF-mitogen-activated protein kinase (MEK)-extracellular signal regulated kinase (ERK) is well regarded as a classical tumor signaling pathway. First, RAS proteins with GTPase activity are activated by upstream RTK, activated RAS activates RAF by binding to the N terminal domain of RAF, activated RAF can further bind to downstream MEK proteins and then activate MEK, activated MEK further activates the downstream unique substrate ERK, and finally activated ERK enters the nucleus, and then causes a series of physiological and biochemical reactions [18]. Additionally, the RAS/ERK pathway represents a canonical mitogen-activated protein kinase (MAPK) pathway, functioning as a key signaling pathway in the regulation of normal cell proliferation, survival, growth, and differentiation [19]. It is also noteworthy that the RAS/ERK pathway is abnormally activated in cancer, wherein the pathway is associated with promoted cell proliferation, malignant transformation, and drug resistance [20]. Besides, there is evidence highlighting the involvement of DIRAS3 in the biological function of the RAS/ERK pathway by increasing the internalization and degradation of epidermal growth factor receptors [21]. In addition, the RAS/extracellular-regulated kinase (ERK) pathway has been extensively studied for several decades, such that this pathway can serve as a potential target for molecule-based pharmacological intervention, and also a small-molecule inhibitor in clinical use [22]. Meanwhile, the RAS/ERK pathway is aberrantly activated in various cancers, contributing to cell proliferation, malignant transformation, as well as drug resistance [20]. Moreover, prior studies have reported that DIRAS3 inhibits the RAS/ERK pathway through increasing internalization and degradation of the epidermal growth factor receptor [21]. Therefore, the current study sets out to explore whether the DIRAS3 gene could influence NSCLC migration and invasion through the RAS/ERK pathway. We adopted A549 cells or H520 cells to investigate the relationship between DIRAS3 and NSCLC and uncovered the inhibitory effect of DIRAS3 over-expression on cell migration and invasion, highlighting DIRAS3 as an effective target for the treatment of NSCLC.

## Materials and methods

### Ethics statement

The current study was approved by the Institutional Ethics Committee of the First Affiliated Hospital of Nanchang University and complied with the standards of the *Declaration of Helsinki*. All operations involving animals were performed in strict accordance with protocols approved by the Animal Care and Use Committee of the First Affiliated Hospital of Nanchang University. Extensive efforts were undertaken to minimize the number and suffering of the experimental animals.

### Patients and sample collection

The NSCLC tissues and adjacent normal tissues were collected from 25 NSCLC patients who had undergone radical surgery at the First Affiliated Hospital of Nanchang University between 2020 and 2021 (the clinical characteristics of NSCLC patients are listed in Table 1). The collected tissues were paraffinized for subsequent experiments.

**Table 1.** Relationship between clinical characteristics and DIRAS3 protein expression in 25 patients with non-small cell lung cancer

Characteristics	Case (n, %)	DIRAS3 protein	p value
**Age** (years)	Media: 61; Range: 42–71		0.247
< 60	12 (48.0)	0.31 ± 0.23	
≥ 60	13 (52.0)	0.21 ± 0.18	
**Gender**			0.260
Male	15 (60.0)	0.22 ± 0.22	
Female	10 (40.0)	0.32 ± 0.19	
**Pathologic type**			0.211
Adenocarcinoma	14 (56.0)	0.30 ± 0.23	
Squamous cell carcinoma	8 (32.0)	0.15 ± 0.14	
Others	3 (12.0)	0.32 ± 0.20	
**Differentiated degree**			0.004
Well	6 (24.0)	0.45 ± 0.26	
Media	11 (44.0)	0.26 ± 0.16	
Poor	8 (32.0)	0.10 ± 0.08	
**TNM stage**			0.007
I–II	16 (64.0)	0.34 ± 0.21	
III–IV	9 (36.0)	0.11 ± 0.11	
**Lymph nodes metastasis**			0.019
No	15 (60.0)	0.33 ± 0.22	
Yes	10 (40.0)	0.14 ± 0.12	

### Cell line source and culture

The human lung adenocarcinoma cell lines (A549, H1299, HCC827, and PC-9) and human lung squamous cell carcinoma cell lines (H520 and H1270) were procured from the American Type Culture Collection (Manassas, VA, USA). Additionally, the normal human bronchial epithelial cell-line BEAS-2B was purchased from Shanghai Cancer Institute (Shanghai, China). The obtained cell lines were identified by short tandem repeats prior to distribution. Subsequently, the cell lines were cultured in Roswell Park Memorial Institute 1640 medium (GIBCO, Grand Island, New York, USA) which comprised of 10% fetal bovine serum (FBS, GIBCO), 100 units/mL penicillin and 100 μg/mL streptomycin, and then cultured in a humidified incubator at 37°C with 5% CO_2_ in air [23].

### Small interfering RNA (siRNA) and DNA transfection and construction of stable cell lines

The aforementioned cells were allowed to achieve 50% confluence prior to siRNA transfection. In accordance with the manufacturer’s protocols, DIRAS3 siRNA, or nonspecific control siRNA were transfected into HCC827 cells using the siLentFect lipid reagent (Bio-Rad, CA, USA). CMV6-DIRAS3-AC-GFP or vector plasmid pCMV6-AC-GFP expression plasmids were purchased from Shanghai GenePharma Co., Ltd. (Shanghai, China). Next, the pCMV6-DIRAS3-AC-GFP plasmid and pCMV6-AC-GFP vector were transfected into A549 cells with the Lipofectamine 2000 reagent (Invitrogen, Carlsbad, CA, USA) as per the manufacturer’s protocols [24].

The DIRAS3 expression vector and control vector packaged with lentivirus were adopted to infect the DIRAS3 over-expressing-A549 cell line (DIRAS3-A549) and control A549 cell line (vector-A549) (GenePharma), respectively. The target cells were infected with lentivirus for a period of 48 hours, and then screened with Purinomyces (Santa Cruz Biotechnology, CA, USA) for 3 weeks.

### PD-0325901 (PD901)-treated cells

In order to test the effect of the RAS/ERK pathway, HCC827 cells were treated with the RAS/ERK pathway inhibitor PD-0325901 (10 mM, dissolved in Dimethyl Sulfoxide [DMSO]) following knockdown of DIRAS3, while the control cells were treated with DMSO. PD-0325901 is a selective inhibitor of two MEK subtypes (MEK1/MEK2), which is capable of preventing the activation of ERK [25–27], and was procured from Pfizer Pharmaceutical Co., Ltd. (Changsha, Hunan).

### Reverse transcript quantitative polymerase chain reaction (RT‑qPCR)

Total RNA content was isolated from the cell lines using the TRIzol RNA isolation reagent (Life Technologies, Carlsbad, CA, USA) and then reverse transcribed into cDNA with the help of cDNA synthesis kits (Takara, Shiga, Japan). RT-qPCR was carried out to detect the mRNA expression patterns of genes using SYBR Premix Ex Taq (Takara) [24]. All RT-qPCR experiments were performed on the GeneAmp® PCR System 9700 (Applied Biosystems, Foster City, CA) using the 7300 real-time PCR system and SDS RQ research software (Applied Biosystems). With glyceraldehyde phosphate dehydrogenase (GAPDH) serving as the internal control, the 2^−ΔΔCt^ method was adopted to detect the relative expression level, and the experiment was repeated 3 times to obtain the mean value. The primer sequences are shown as follows: DIRAS3: (forward) 5′-CCCGCCCTGCTTATCCT-3, (reverse) 5′-CGTCGCCACTCTTGCTGT-3′; GAPDH: (forward) 5′-GAAGGTGAAGGTCGGAGTC-3′, (reverse) 5′-GAAGATGGTGATGGGATTTC-3′.

### Western blot assay

Western blot assay and immunoprecipitation were carried out as described in the previously published literature [28]. Briefly, tissues and cells were lysed in a radioimmunoprecipitation assay buffer (Beyotime, Shanghai, China) containing protease inhibitors. Subsequently, the total protein content was dissolved using sodium dodecyl sulfate polyacrylamide gel electrophoresis and transferred onto a polyvinylidene fluoride membrane (Millipore, Billerica, MA, USA) which was blocked with 5% milk Tris-buffered saline plus 0.1% Tween-20 (TBST) at room temperature for 1 hour. Next, the membrane was incubated with the primary antibody at 4°C overnight and the secondary antibody (Goat Anti-Mouse IgG HRP Conjugate (H + L) antibody, Millipore Cat# 71045–3, RRID: AB_11211441, dilution ratio of 1:5000) at room temperature for 1 hour. Enhanced chemiluminescence reagent (Beyotime) was utilized to detect the band, and β-actin was adopted as the internal reference. The aforementioned primary antibodies included the following: mouse anti-DIRAS3 (LifeSpan Cat# LS-C136801-100, RRID: AB_10945080, dilution ratio of 1:1000), mouse anti-E-cadherin (Cell Signaling Technology Cat# 14472, RRID: AB_2728770), mouse anti-N-cadherin (Cell Signaling Technology Cat# 14215, RRID: AB_2798427), mouse anti-Vimentin (Cell Signaling Technology Cat# 3932, RRID: AB_2288553), mouse anti-RAS (Santa Cruz Biotechnology Cat# sc-30, RRID: AB_627865, dilution ratio of 1:500), mouse anti-ERK (LifeSpan Cat# LS-C16335-100, RRID: AB_862662, dilution ratio of 1:2000), mouse anti-p-ERK (Santa Cruz Biotechnology Cat# sc-81492, RRID: AB_1125801, dilution ratio of 1:1000) and mouse anti-β-actin (Santa Cruz Biotechnology Cat# sc-47778, RRID: AB_626632, dilution ratio of 1:1000).

### Transwell assay

A modified double chamber plate (Corning, New York, NY, USA) with an aperture of 8 μm was adopted for cell migration and invasion test. Transwell membranes with or without Matrigel (BD Biosciences, Mississauga, Canada) were utilized for invasion and migration test. In accordance with the previously published literature [29], the transfected HCC827 and A549 cells were harvested, suspended in a serum-free medium, and placed in the upper chamber for migration or invasion analysis, respectively. Meanwhile, a medium supplemented with 10% FBS was placed in the lower chamber. After 24 hours of culture, the cells migrated through the membrane or invaded the lower surface were fixed, stained, and counted under an inverted microscope (Olympus, Tokyo, Japan).

### In vivo *metastasis assay*

Male BALB/c nude mice (aged 4–5 weeks, weighing 20–22 g) were purchased from Shanghai SLAC Experimental Animal Co., Ltd. (Shanghai, China) and housed at the Animal Experimental Center of the First Affiliated Hospital of Nanchang University under specific pathogen-free (SPF) conditions. The BALB/c nude mice were randomly divided into the DIRAS3 group and the vector group, with six mice in each group. DIRAS3-A549 cells or vector-A549 cells (2 × 10^6^) were injected into the tail vein of nude mice in 200 μL phosphate buffered saline. Two months later, mice in both groups were euthanized. Subsequently, the lung tissues were removed and fixed with 10% formalin. Calculation of the number of metastatic nodules and histopathological analyses were carried out [30]. A number of metastatic nodules on the surface of the lung were observed using a stereo microscope (S8 APO, Leica Microsystems, Wetzlar, Germany).

### Statistical analysis

Statistical analyses were performed using the Prism 8.0 software (GraphPad, La Jolla, CA, USA). Student’s t-test was adopted for comparisons between two groups and the one-way analysis of variance (ANOVA) and Tukey’s post hoc test were utilized for comparisons among multiple groups. A value of *p* < 0.05 was considered to be statistically significant.

## Results

### DIRAS3 is poorly expressed in NSCLC cells

**Figure 1.**
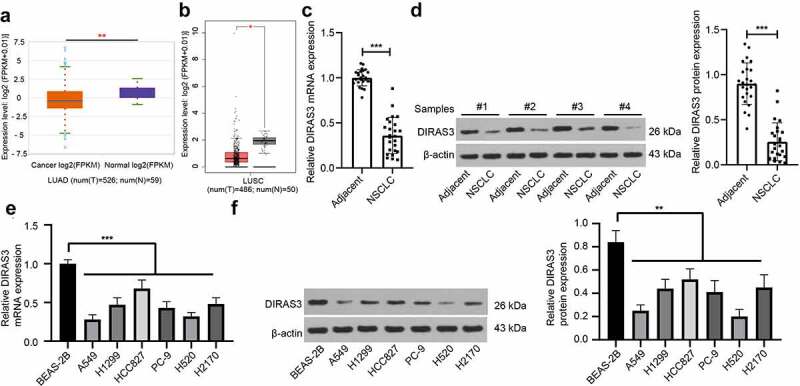
DIRAS3 is lowly expressed in NSCLC cells. A-B. DIRAS3 expression in lung adenocarcinoma (a) and lung squamous cell carcinoma (b) through online database. C-D. The mRNA and protein expression of DIRAS3 in NSCLC tissues were determined RT-qPCR (c) and Western blot assay (d) (n = 25). E-F. The mRNA expression of DIRAS3 in NSCLC cell lines (A549, H1299, HCC827, PC-9, H520 and H2170) and normal lung epithelial cell line BEAS-2B were determined RT-qPCR (e) and Western blot assay (f). *P < 0.05, **P< 0.01, ***P < 0.001. The experiment was repeated three times and the data were expressed as mean ± standard deviation. Paired t-test, One-way ANOVA and Tukey’s post hoc test were used for analysis.

Existing evidence indicates that DIRAS3 is capable of inhibiting tumor growth in various cancers [24,31–33], yet its role and mechanism in regard to NSCLC remains elusive. To first explore the expression pattern of DIRAS3 in NSCLC, we first searched the StarBase database (http://starbase.sysu.edu.cn/panGeneDiffExp.php) and the GEPIA database (http://gepia.cancer-pku.cn/detail.php?gene=DIRAS3&clicktag=boxplot) and found that DIRAS3 was poorly expressed in lung adenocarcinoma and lung squamous cell carcinoma (Figure 1(a,b)). Subsequently, we detected DIRAS3 expression patterns in NSCLC tissues and adjacent normal tissues of patients with NSCLC with the help of RT-qPCR and Western blot assay. The findings demonstrated low expression levels of DIRAS3 in NSCLC tissues of patients with NSCLC relative to those in adjacent normal tissues (Figure 1(c,d)). We further analyzed the relationship among DIRAS3 protein expression and age, gender, pathological grade, pathological differentiation, TNM stage, and lymph node metastasis (LNM) of NSCLC patients, and as illustrated in Table 1, we uncovered that DIRAS3 protein expression was associated with pathological differentiation, TNM stage, and LNM. Furthermore, we quantified DIRAS3 expression patterns in different NSCLC cell lines, which revealed that DIRAS3 expression were diminished in A549, H1299, HCC827, PC-9, H520, and H2170 cells versus BEAS-2B cells (Figure 1(e,f)). Accordingly, we selected the human lung adenocarcinoma cell lines (A549 and HCC827) and human lung squamous cell carcinoma cell lines (H520 and H2170) for follow-up experiments.

### DIRAS3 suppresses NSCLC cell migration and invasion

**Figure 2.**
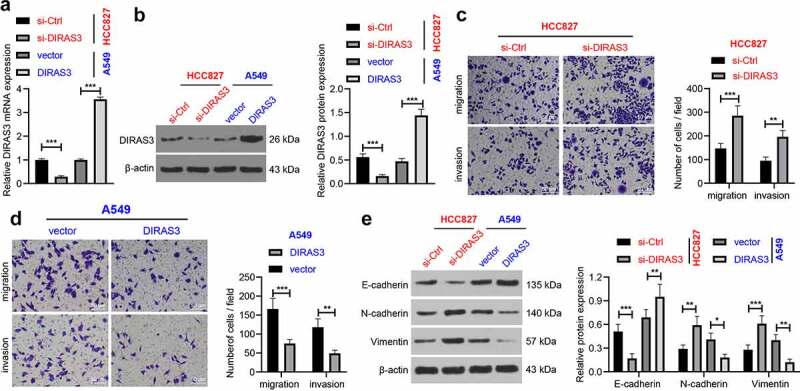
Overexpression of DIRAS3 inhibits NSCLC (HCC827 and A549) cell migration and invasion *in vitro*. A-B. The mRNA expression of DIRAS3 in NSCLC cells with different treatments were determined RT-qPCR (a) and Western blot assay (b). C-D. Transwell assay was used to detect the migration and invasion ability of HCC827 cells with DIRAS3 knockdown (c) or A549 cells with DIRAS3 overexpression (d). E. Expression of EMT-related proteins in HCC827 cells with DIRAS3 knockdown or A549 cells with DIRAS3 overexpression. *P < 0.05 **P< 0.01, ***P < 0.001. The experiment was repeated three times and the data were expressed as mean ± standard deviation. Student’s t was used for analysis.

**Figure 3.**
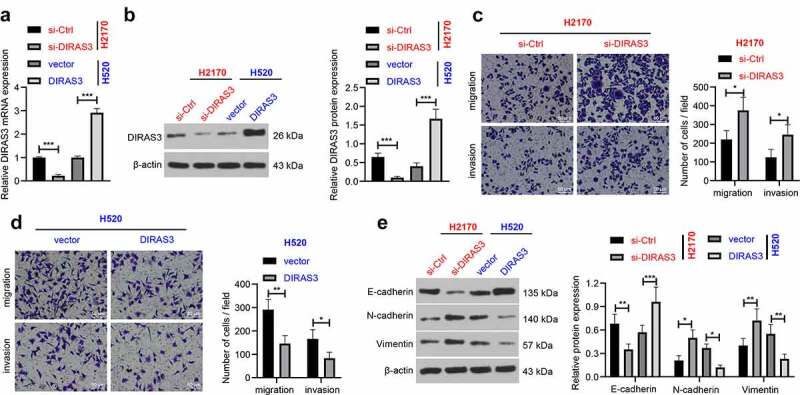
Overexpression of DIRAS3 inhibits NSCLC (H2170 and H520) cell migration and invasion *in vitro*. A-B. The mRNA and protein expression of DIRAS3 in NSCLC cells (H2170 and H520) with different treatments were determined RT-qPCR (a) and Western blot assay (b). C-D. Transwell assay was used to detect the migration and invasion abilities of H2170 cells with DIRAS3 knockdown (c) or H520 cells with DIRAS3 overexpression (d). E. Expression of EMT-related proteins in H2170 cells with DIRAS3 knockdown or H520 cells with DIRAS3 overexpression. **P< 0.01, ***P < 0.001. The cell experiment was repeated three times and the data were expressed as mean ± standard deviation. Student’s t was used for analysis.

Next, in order to further investigate the role of DIRAS3 in the migration and invasion of NSCLC cells, we silenced or over-expressed DIRAS3 in human lung adenocarcinoma cell lines (HCC827 and A549), respectively. After 48 hours of transfection, DIRAS3 mRNA and protein expression levels were significantly knocked-down or over-expressed in NSCLC cells, respectively (Figure 2(a,b)). In addition, the results of Transwell cell migration and invasion assay that DIRAS3 knockdown enhanced NSCLC cell migration and invasion in the HCC827 cell line (Figure 2(c)). In contrast, in A549 cells, over-expression of DIRAS3 brought about inhibition of NSCLC cell migration and invasion (Figure 2(d)). Furthermore, we detected the expression patterns of epithelial-mesenchymal transition (EMT)-related proteins by means of a Western blot assay. It was found that knockdown of DIRAS3 contributed to down-regulation of E-cadherin, and up-regulation of N-cadherin and Vimentin in HCC827 cells, whereas DIRAS3 over-expression resulted in the opposite trends in A549 cells (Figure 2(e)). Similarly, we silenced or over-expressed DIRAS3 in human lung squamous cell carcinoma cell lines (H520 and H2170), and came across similar results (Figure 3). Together, these findings indicated that over-expression of DIRAS3 inhibits NSCLC cell migration and invasion *in vitro*.

### *DIRAS3 inhibits metastasis of NSCLC* in vivo

**Figure 4.**
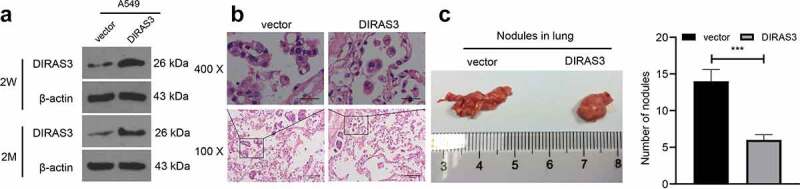
Overexpression of DIRAS3 significantly inhibits the metastasis of NSCLC cells *in vivo*. A. Western blot assay was utilized to test DIRAS3 protein expression in vector-A549 cell lines and DIRAS3-A549 cell lines. B. Two months after tail vein injection of DIRAS3-A549 cell line, the lung tissues were observed with HE staining. C. Representative images of lung metastatic nodules (arrows indicate metastatic nodules) fixed by 10% formalin buffer and counts of lung metastatic nodules two months after the injection of vector-A549 and DIRAS3-A549 stable cell lines. n = 6. ***P < 0.001. The data were expressed as mean ± standard deviation. Student’s t was used for analysis.

Additionally, we explored whether DIRAS3 could inhibit the metastasis of NSCLC *in vivo* to validate our preceding findings. DIRAS3-A549 and vector-A549 cell lines were established. Two weeks after the lentivirus infection, DIRAS3 protein levels in the cell lines were detected with the help of a Western blot assay. Subsequently, the cells were incubated for 2 months without adding puromycin, which revealed that DIRAS3 protein expression levels did not change in the two stable cell lines (Figure 4(a)). The BALB/c nude mice were randomly divided into the DIRAS3 group and the vector group (six mice in each group), and these nude mice were injected with DIRAS3-A549 and vector-A549 cells *via* the tail vein. Two months later, nude mice in both groups were euthanized and the lungs were removed and fixed with 10% formalin. Quantification of the number of metastatic nodules and further histopathological examination were performed. The selected metastatic nodules were verified by HE staining (Figure 4(b)). In contrast to the vector group, the detectable tumor nodules in the lungs of the DIRAS3 group grew slower, and the number of tumor nodules was significantly reduced (Figure 4(c)). Overall, these findings suggest that over-expression of DIRAS3 significantly inhibits the metastasis of NSCLC cells *in vivo*.

### DIRAS3 inhibits the activation of RAS/ERK pathway to inhibit NSCLC cell migration and invasion

**Figure 5.**
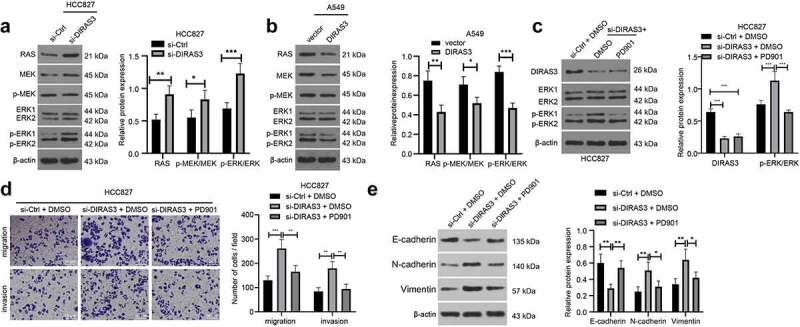
DIRAS3 blocks the activation of Ras/ERK pathway to restrict the migration and invasion of NSCLC cells. A-B. Western blot assay was used to detect the expression of RAS/ERK pathway-related proteins in HCC827 cells after DIRAS3 knockdown (a) or A549 cells overexpression (b). C. The protein expression levels of DIRAS3 and RAS/ERK pathway-related proteins in NSCLC cells with different treatments were determined by Western blot assay. D. Transwell assay was used to detect the migration and invasion ability of HCC827 cells with DIRAS3 knockdown and RAS/ERK pathway inhibitor PD901. E. The protein expression levels of EMT-related proteins in NSCLC cells with different treatments were determined by Western blot assay. *P< 0.05, **P< 0.01, ***P < 0.001. The experiment was repeated three times and the data were expressed as mean ± standard deviation. Student’s test, One-way ANOVA and Tukey’s post hoc test were used for analysis.

Accumulating evidences have shown that RAS/ERK pathway plays a role in cancer cell metastasis [34,35]. In addition, prior studies have indicated that DIRAS3 can inhibit the RAS/ERK pathway [21]. Accordingly, we explored whether DIRAS3 affected the expression of the RAS/ERK pathway in NSCLC, and the results of the Western blot assay illustrated that knockdown of DIRAS3 in HCC827 cells increased RAS and p-ERK/ERK expression levels (Figure 5(a)), while over-expression of DIRAS3 in A549 cells decreased RAS and p-ERK/ERK expression levels (Figure 5(b)). Together, these findings suggest that DIRAS3 could inhibit the activation of RAS/ERK pathways in NSCLC cells.

To further validate that DIRAS3 regulates the RAS/ERK pathway in NSCLC cells, we treated the cells with a 10 mM RAS/ERK pathway inhibitor PD901 in HCC827 cells following the silencing of DIRAS3 (Figure 5(c)). Subsequent results from transwell cell migration and invasion experiments revealed that in the HCC827 cell line, cell migration and invasion abilities were enhanced following DIRAS3 knockdown while undergoing a reduction after further treatment with the RAS/ERK pathway inhibitor PD901 (Figure 5(d)). Additionally, a Western blot assay was carried out to detect the expression patterns of EMT-related proteins to further illustrate this phenomenon from the molecular level (Figure 5(e)). Collectively, these findings indicate that DIRAS3 may inhibit the migration and invasion of NSCLC cells by modulating the RAS/ERK pathway.

## Discussion

The last few years have witnessed a surge in novel biomarker-directed treatments for NSCLC due to our enhanced understanding and recognition of both the biological and molecular subtypes of NSCLC. Newer empirical treatment regimens, in conjunction with these biomarker-directed therapies, have greatly improved the overall survival of metastatic NSCLC patients [4]. Nevertheless, the overall 5-year survival rate of patients remains dismal despite the monumental progress achieved with regard to targeted therapy and novel immunotherapy. In lieu of the same, it is prudent to further probe into the molecular mechanisms implicated in NSCLC development to advance therapeutic regimens against NSCLC. Herein, findings uncovered in our study reveal that DIRAS3 may play a role in the migration and invasion of NSCLC *via* inhibition of the RAS/ERK pathway.

There is a plethora of evidence suggestive of the inhibitory role conferred by DIRAS3 on tumor growth of various cancers [24,31–33], however, but its potential role and mechanism in NSCLC remain to be unknown. Herein the current study, we first detected the DIRAS3 expression pattern in NSCLC tissues and found that DIRAS3 was poorly expressed in NSCLC tissues. In addition, we quantified DIRAS3 expression levels in several human lung adenocarcinoma cell lines (A549, H1299, HCC827, and PC-9) and human lung squamous cell carcinoma cell lines (H520 and H1270), and subsequent results illustrated that DIRAS3 expressions were diminished in A549, H1299, HCC827, PC-9, H520, and H2170 cells compared to those in BEAS-2B cells. Accordingly, we have selected the aforementioned human lung adenocarcinoma cell lines (A549 and HCC827) and human lung squamous cell carcinoma cell lines (H520 and H2170) for follow-up experiments. Thereafter, we investigated the effects of DIRAS3 on the migration and invasion of NSCLC cells, and on the metastasis of NSCLC *in vivo*. Our findings demonstrate that over-expression of DIRAS3 can inhibit NSCLC cell migration and invasion *in vitro* and also reduce the metastasis of NSCLC cells *in vivo*. In line with our findings, Jingping Qiu *et al*. documented that up-regulation of DIRAS3 suppressed metastatic foci formation in the lung and liver during the process of proliferation, anti-apoptosis, and angiogenesis, as well as survival in the vasculature. Moreover, the preceding study also stated that DIRAS3 over-expression also suppressed extracellular matrix degradation, and further diminished cell migration and invasion in gastric cancer [24]. Meanwhile, the study performed by Zou *et al*. suggested that the up-regulation of DIRAS3 suppressed cell cycle arrest at the G0/G1 stage while inducing cell cycle arrest at the G2/M stage in ovarian and breast cancers [12]. Also, the restoration of DIRAS3 was previously shown to suppress cell proliferation and stimulate autophagy in ovarian cancer [31,36]. Supportedly, the upregulated DIRAS3 has been demonstrated to restrict lung cancer cell proliferation and invasion while inducing apoptosis [16]. Further, in accordance with our findings, Chen J *et al*. previously indicated that DIRAS3 over-expression leads to attenuation of migration and invasion *via* inhibition of glioma cell growth [37]. Furthermore, our findings illustrate that DIRAS3 inhibits EMT suppress NSCLC cell migration and invasion, which is in accordance with findings uncovered by Ouyang J *et al.* [38].. Moreover, the study carried out by Xiaohong Wu *et al*. supported that over-expression of DIRAS3 might be associated with the inhibition of lung cancer cell proliferation and invasion [16].

A growing number of studies have suggested that the RAS/ERK pathway plays a role in cancer cell metastasis [34,35]. Interestingly, there is prior evidence to suggest that DIRAS3 can inhibit the RAS/ERK pathway [21]. In our study, the obtained findings indicate that DIRAS3 retarded the activation of the RAS/ERK pathway in NSCLC cells. Similarly, previous studies have conjectured that activating the RAS/ERK pathway can induce tumor cell proliferation and inhibit apoptosis, whereas inhibition of the said pathway is associated with reduced tumor development [39,40]. Meanwhile, abnormalities in the RAS/ERK pathway have also been revealed to promote the development of NSCLC [41,42]. The above-mentioned evidences indicate the ability of the RAS/ERK pathway to serve as a cancer-inducing agent in the carcinogenesis of various cancers. Furthermore, we explore whether DIRAS3 regulated the RAS/ERK pathway in NSCLC cells, and our findings suggest that DIRAS3 may inhibit NSCLC cell progression by modulating the RAS/ERK pathway. In accordance with our findings, the study performed by Lu Z *et al*. demonstrated that DIRAS3 inhibited the RAS/ERK pathway by augmenting the internalization and degradation of epidermal growth factor receptors [21]. Meanwhile, Sutton MN *et al*. previously highlighted that DIRAS3 can directly form a heteromeric interaction with RAS, wherein disrupting RAS aggregation and blocking Raf kinase activation, thereby inhibiting RAS/MAPK signaling to inhibit cancer cell growth [37]. Additionally, prior studies have indicated that DIRAS3 interacts with C-RAF and down-regulates MEK activity to limit cell migration [38]. Altogether, the aforementioned findings and evidence indicate that DIRAS3 inhibits the activation of the RAS/ERK pathway in NSCLC cells, thereby inhibiting NSCLC cell migration and invasion. However, the underlying mechanism behind the DIRAS3-related suppression of NSCLC migration and invasion *via* inhibition of the RAS/ERK pathway requires further exploration.

## Conclusions

In summary, the current study suggests that up-regulation of DIRAS3 in NSCLC inhibits tumor proliferation and invasiveness by suppressing the RAS/ERK pathway. Our findings indicate that DIRAS3 plays an inhibitory role in NSCLC, and further highlight the potential of DIRAS3 as a larval diagnostic marker and novel therapeutic target for NSCLC. Nevertheless, the current study solely explores the effects of DIRAS3 on NSCLC cell migration and invasion, whereas multiple studies have implicated DIRAS3 in autophagy signaling, thus how DIRAS3 will participate in autophagy in NSCLC is also worth investigating. Moreover, it would be prudent to further explore the relationship between DIRAS3 expression and its migration and invasion abilities in different types of lung cancer cells to fully utilize the diagnostic and therapeutic roles of DIRAS3 in NSCLC.

## Data Availability

At this time, individual participant data have not been made available. However, reasonable requests made to the corresponding author for de-identified patient-level data that underlie the results reported in this article will be considered.
